# Detection of gas bubbles and local voids in molecular simulations using *burbuja*

**DOI:** 10.1002/pro.70562

**Published:** 2026-04-21

**Authors:** Abraham Muñiz‐Chicharro, Lane W. Votapka, Rommie E. Amaro

**Affiliations:** ^1^ Department of Molecular Biology University of California San Diego La Jolla California USA

**Keywords:** bubbles, equilibration, error detection, explicit solvent, molecular dynamics, validation, voids

## Abstract

We present *burbuja* (Baring Unseen Regions of Bubbles Using Joint‐Density Analysis), an automated software tool for detecting and characterizing gas bubbles and other local voids in molecular structures and trajectories containing explicit aqueous solvent. We describe the *burbuja* algorithm and demonstrate its accuracy and utility across a range of example systems, including globular proteins, a membrane system, a coarse‐grained membrane trajectory with 25,000 frames, a large envelope capsid containing approximately 150 million atoms, and a very large respiratory aerosol system containing approximately 1 billion atoms. *Burbuja* supports optional GPU acceleration and can be run as a standalone command‐line utility or through a Python‐based API, facilitating integration with existing community tools for molecular system preparation and analysis. *Burbuja* is open‐source and freely available at https://github.com/Abrahammc90/Burbuja.git.

## 1 INTRODUCTION

Molecular dynamics (MD) simulations have become a cornerstone of molecular research, enabling the study of atomic motions and interactions through classical representations of interatomic forces. One of the most widely used and accurate approaches employs *explicit solvent* models, in which solvent molecules such as water and electrolyte ions are represented as individual point particles (Levy & Gallicchio, 1998; van der Spoel et al., 2022). In contrast, *implicit solvent* simulations include only the solute particles and approximate solvent effects through mathematical equations, trading some accuracy for increased computational efficiency (Onufriev, 2010; Severoglu et al., 2025). In this work, we focus exclusively on explicit solvent simulations. In our experience and opinion, unwanted bubbles are not going to be a problem for systems that employ typical implicit solvent methods.

A common issue in explicit MD simulations is the unintended formation of *bubbles*—regions of low or zero solvent density. While bubbles can sometimes be introduced intentionally for specific studies (Aluthgun Hewage & Meegoda, 2022; Min & Berkowitz, 2019), accidental bubble formation in periodic explicit solvent simulations can severely compromise the realism of liquid‐state systems. Such artifacts are encountered by both novice and experienced researchers. In our experience, bubbles can arise from incorrectly defined periodic box vectors or from faulty heating or equilibration procedures that cause the formation of unwanted cavities within the solvent. Large and complex systems are particularly prone to bubble formation, as the assembly, solvation, and equilibration of multiple substructures can easily produce voids.

Once present, bubbles in explicit solvent systems can persist for long simulation times. Although their presence may not immediately destabilize the simulation numerically, they waste computational resources and can lead to invalid or misleading results. Visualization software can reveal such bubbles, but manual inspection becomes tedious when analyzing many trajectories or very large systems.

Numerous powerful software tools exist for the general analysis of molecular structures and trajectories—such as Travis (Brehm et al., 2020), MDTraj (McGibbon et al., 2015), and MDAnalysis (Gowers et al., 2016). While these frameworks could, in principle, be adapted to detect bubbles automatically, no dedicated implementation currently exists to our knowledge. We are likewise unaware of any tool other than *burbuja* (Baring Unseen Regions of Bubbles Using Joint‐Density Analysis) specifically designed for the detection and characterization of bubbles in molecular simulation structures and trajectories.

Although general‐purpose density analysis and volumetric mapping tools exist within visualization and trajectory‐analysis frameworks such as VMD (VolMap) (Humphrey et al., 1996), MDTraj, and MDAnalysis, these packages do not include automated routines for detecting or characterizing bubble‐like voids in explicit solvent systems. *Burbuja* addresses this gap by providing an automated and scalable approach specifically optimized for identifying and quantifying such regions within molecular simulations.

Here, we present *burbuja*, an automated bubble‐detection tool for MD simulations. *Burbuja* enables researchers to identify bubbles during system preparation or analysis and can be incorporated into automated workflows to detect artifacts in early simulation stages—preventing wasted computational effort and ensuring data validity.

## 2 RESULTS

**FIGURE 1:**
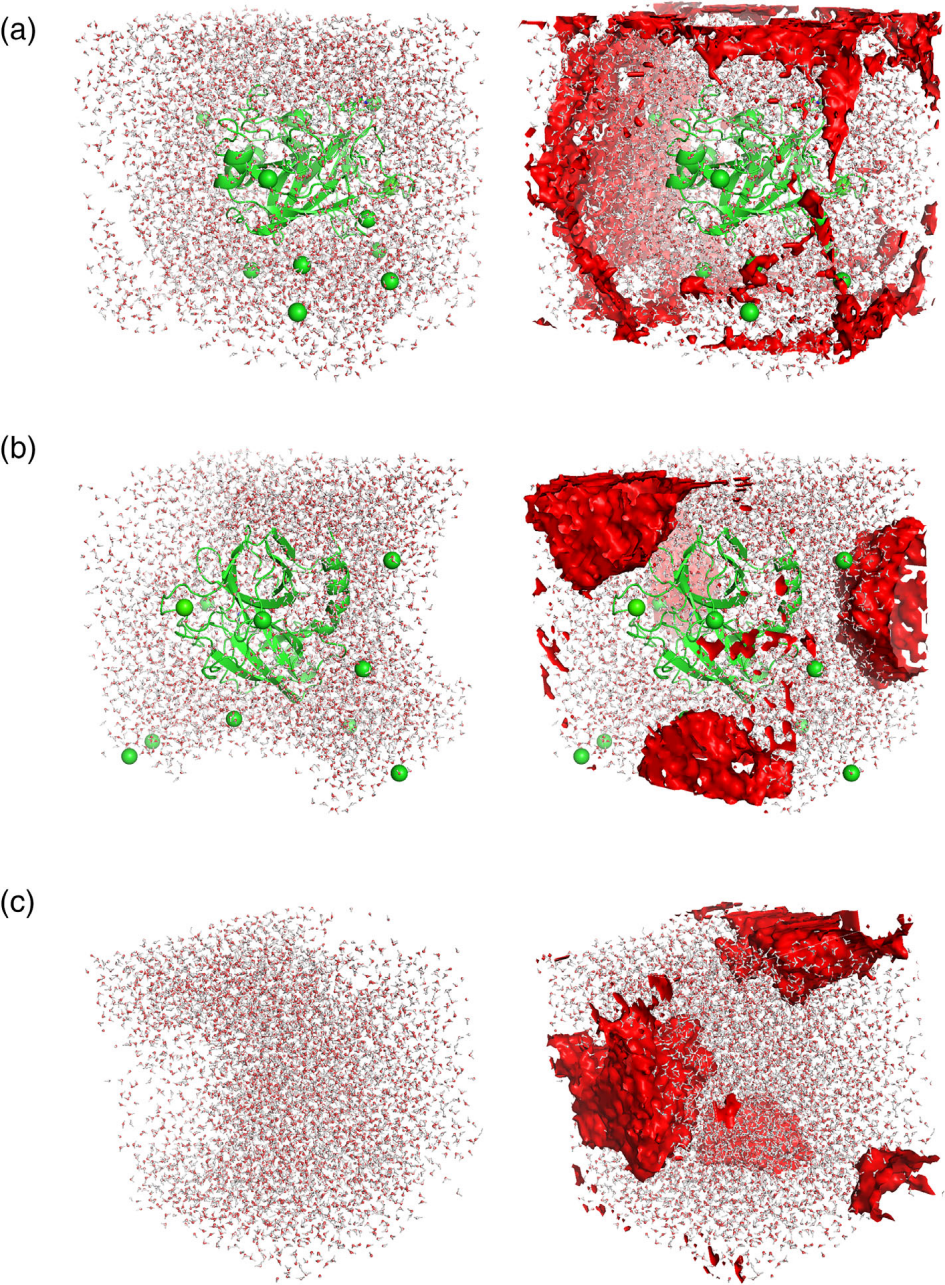
Test systems with voids detected (regions colored in red). In panel (a), a system employing incorrect truncated octahedral periodic box vectors was wrapped, producing an assortment of planar‐shaped voids. In panel (b), a spherical bubble has formed, yet various portions of the bubble have been wrapped into different edges of the box. In panel (c), a solvent‐only system contains a similar spherical bubble.

*Burbuja* successfully identified voids arising from poorly wrapped boxes or insufficient equilibration (Figure 1).

**FIGURE 2:**
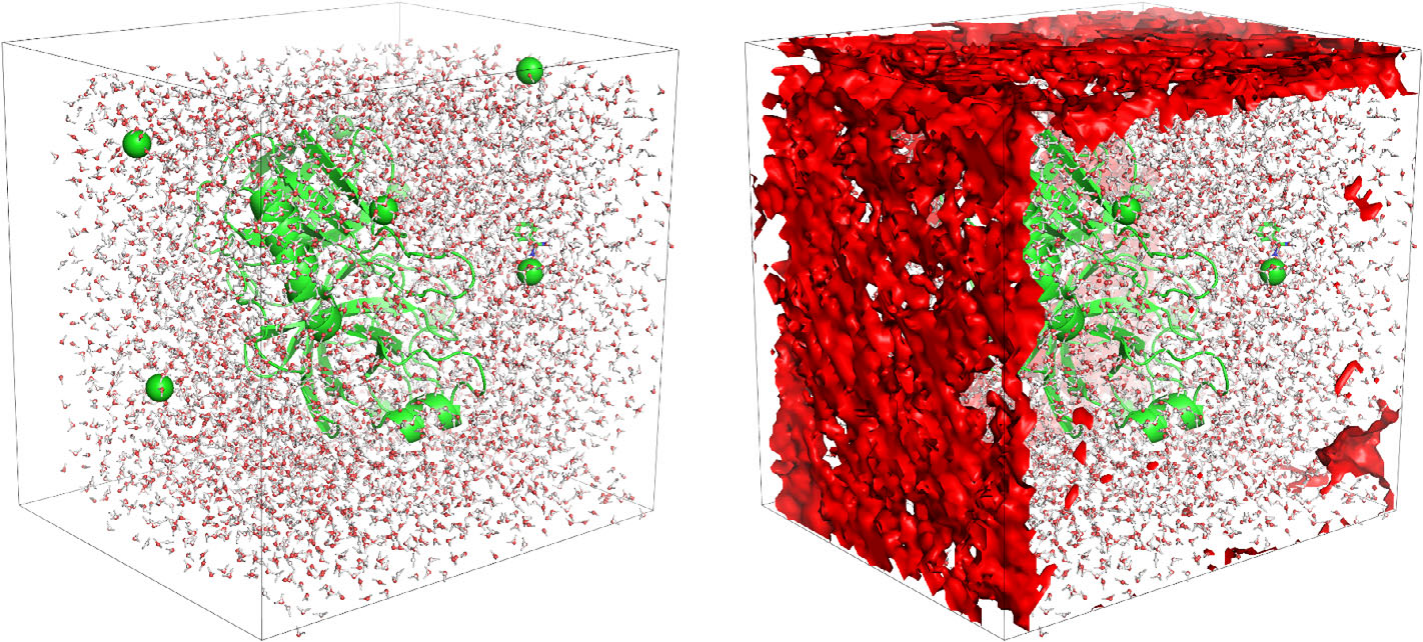
System with incorrect box boundaries. The overly‐large rectangular periodic box produces empty regions along the sides, which were successfully identified by *burbuja* (Baring Unseen Regions of Bubbles Using Joint‐Density Analysis).

To further challenge the method, we analyzed a system with incorrectly defined box boundaries. In this case, *burbuja* correctly detected the affected sides as low‐density regions (Figure 2).

**FIGURE 3:**
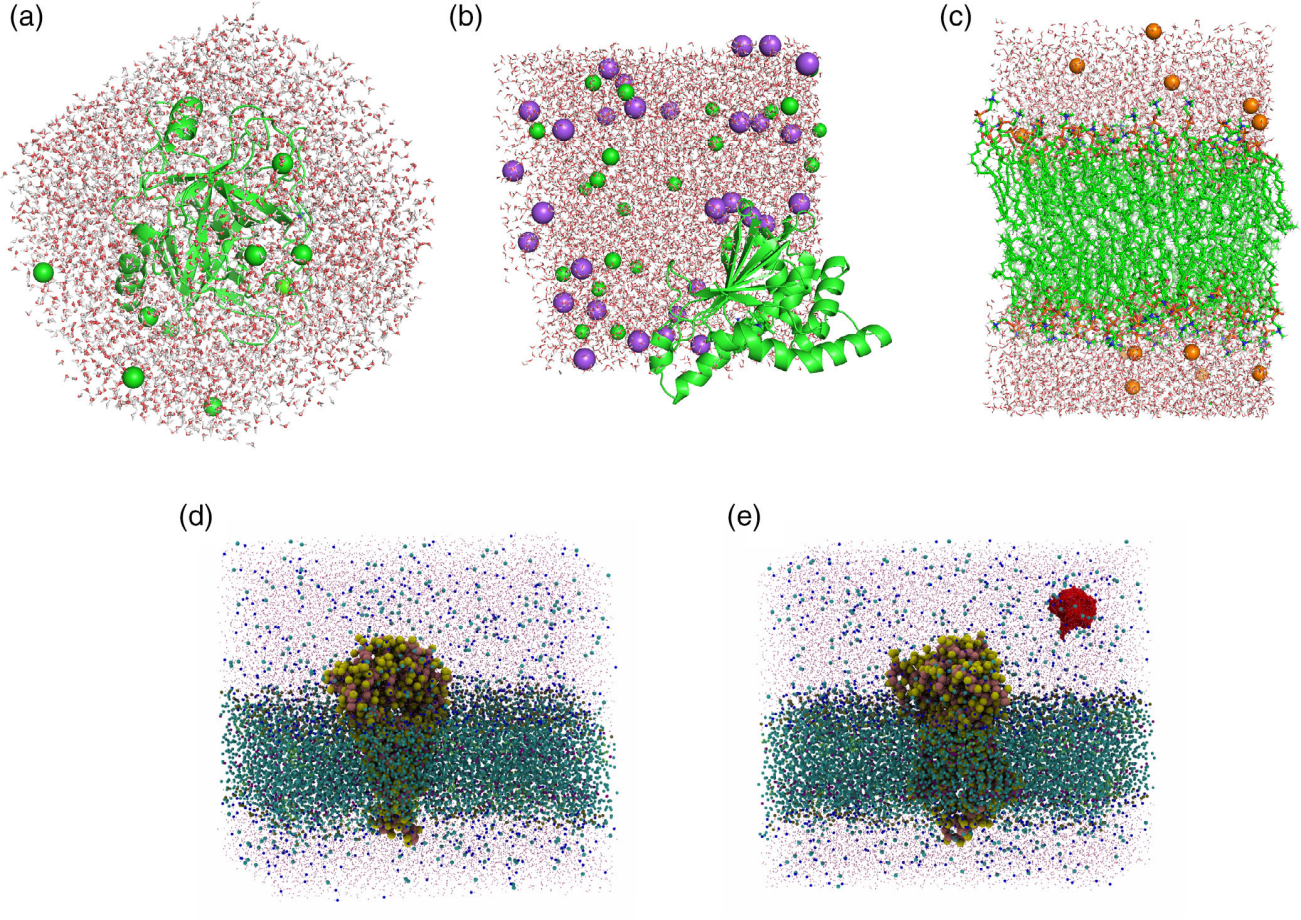
Representative examples of systems analyzed with *burbuja* (Baring Unseen Regions of Bubbles Using Joint‐Density Analysis). (a) Triclinic box containing a solvated protein—showing that *burbuja* does not require rectangular systems. (b) Protein positioned outside the water box can create a void in the adjacent periodic image that resembles a bubble, yet *burbuja* correctly classified this, and detected no false positives. (c) *Burbuja* detects no voids within the membrane system containing low‐density regions within the lipid bilayer. (d) Coarse‐grained system of the SARS‐Cov‐2 M‐protein. (e) Coarse‐grained system of the SARS‐Cov‐2 M‐protein with an artificial void detected by *burbuja* and depicted in red.

We also evaluated *burbuja* on systems prone to false‐positive detections. The method accurately avoided misclassifying legitimate structural regions as voids (Figure 3a–c). In addition to these systems, *burbuja* was tested on a coarse‐grained (CG) simulation trajectory of a membrane system comprised of 31,000 particles and approximately 25,000 frames (Figure 3d,e).

**FIGURE 4:**
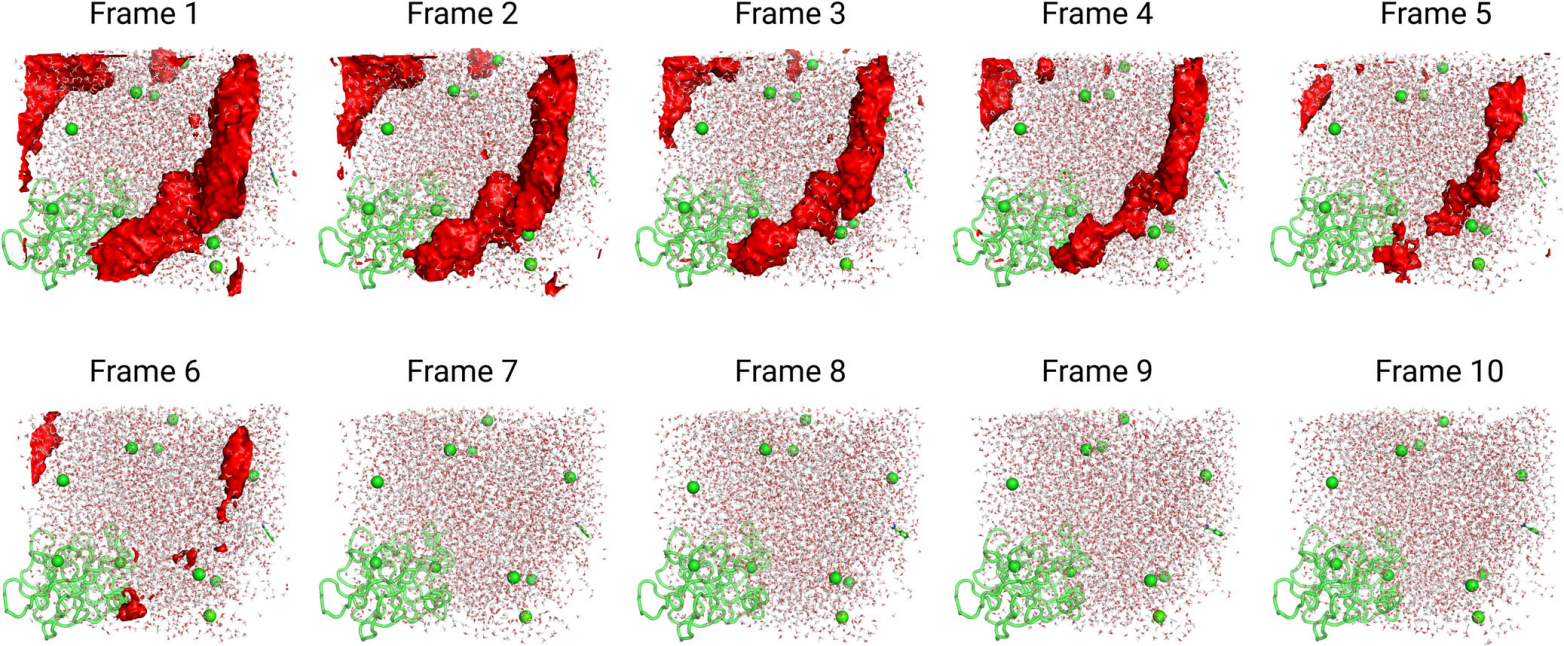
Ten frames from the constant‐pressure molecular dynamics equilibration of the trypsin–benzamidine system were analyzed to track voids over time. *Burbuja* (Baring Unseen Regions of Bubbles Using Joint‐Density Analysis) successfully identified and followed these voids until their disappearance, without misclassifying the periodic image of the protein (apparent void in the lower‐right corner).

In addition to single‐frame analyses, *burbuja* was able to follow the evolution of voids throughout an equilibration trajectory. Notably, it tracked the gradual disappearance of transient voids without falsely reporting a void at the box corner, where the space is actually occupied by the protein through periodic boundary conditions (Figure 4).

**FIGURE 5:**
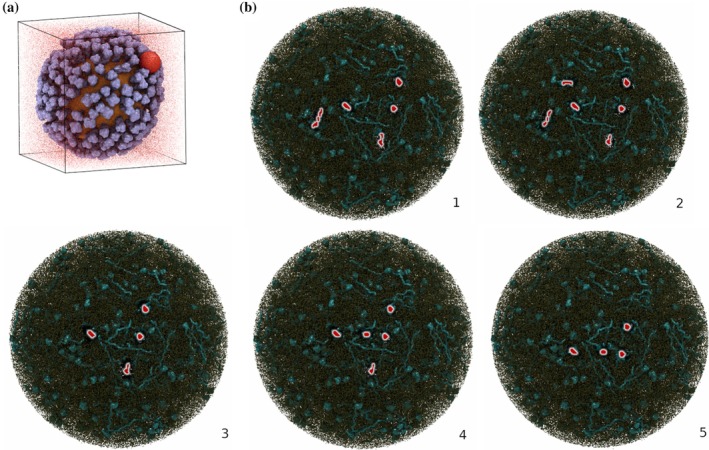
Virus and aerosol systems. (a) Influenza viral membrane system (~150 million atoms) analyzed with *burbuja* (Baring Unseen Regions of Bubbles Using Joint‐Density Analysis). The artificially generated bubble (red sphere) was detected among the glycoproteins (purple) of the viral bilayer (orange). Water molecules are also shown as the red dots to show the water box, although to improve visibility, most of the water molecules are hidden. (b) Respiratory aerosol system equilibration trajectory (~1 billion atoms; five frames) analyzed with *burbuja*. The bubbles (red shapes) are present throughout the equilibration within the aerosol particle, composed of proteins (cyan surfaces), phospholipids (brown spheres), and other particles. To improve visibility within the respiratory aerosol, most molecules are hidden.

*Burbuja* was next applied to a large explicitly solvated molecular system: a simulated influenza virion comprising approximately 150 million atoms (Casalino et al., 2020, 2022). Since the published structure contained no natural voids, we introduced an artificial bubble by removing all solvent molecules within a 5 nm radius around a randomly chosen glycoprotein atom on the viral surface. *Burbuja* accurately detected this spherical bubble nested among the glycoproteins (purple) embedded in the viral bilayer (orange) (Figure 5a). Finally, we also tested *burbuja* on a very large respiratory aerosol system containing approximately 1 billion atoms (Figure 5b). The aerosol system was unusual, since it consisted of a spherical droplet of solvent, surrounded by vacuum, although still within a periodic box. In addition to these large systems, *burbuja* was tested on a CG simulation trajectory of a membrane system comprised of 31,000 particles and approximately 25,000 frames.

### 2.1 Performance evaluation

*Burbuja* demonstrated high computational efficiency, particularly when using graphical processing unit (GPU) acceleration (Table 1). Timing comparisons were performed using both CPU‐only and GPU‐enabled modes on an Nvidia RTX 6000 Ada graphics card. For a medium‐sized system (trypsin–benzamidine, ~23,000 atoms), the GPU achieved a modest speedup (~3.2×) for a single‐frame analysis, and a more significant gain (~7.3×) for a 10‐frame trajectory. For the very large influenza virion (150 million atoms), the GPU acceleration yielded a substantial speedup of approximately 13.3×. The CG single‐frame system experienced a ~12× speedup using the GPU implementation compared to the central processing unit (CPU). The respiratory aerosol system (1 billion atoms) and the long CG trajectory were computable using the GPU, although we did not run it on the CPU for comparison, since the fast GPU implementation sufficed for our purpose.

**TABLE 1:** *Burbuja* (Baring Unseen Regions of Bubbles Using Joint‐Density Analysis) bubble detection times.

System	Size (atoms)	Number of frames	Time using CPU (s)	Time using GPU (s)
Trypsin–benzamidine	~23,000	1	1.9	0.6
Trypsin–benzamidine	~23,000	10	22.5	3.1
Influenza virion	~150 million	1	~16,000	~1200
Respiratory aerosol	~1 billion	1	−/−	~3400
Respiratory aerosol	~1 billion	5	−/−	~13,800
CG membrane system	31,000	1	33.0	2.8
CG membrane system	31,000	25,000	−/−	~57,000

Abbreviation: CG, coarse‐grained.

*Burbuja* is simple to install and use. It can be executed via a single command‐line instruction or accessed as a Python‐based application programming interface (API) for integration into existing molecular preparation or simulation workflows or for analysis with Jupyter notebooks. As for output, by default, *burbuja* will simply indicate whether or not a bubble is present in the provided system. However, if directed, additional information can be produced, including the number of distinct bubbles, the volume of each bubble, and a volumetric map file (in .dx format) indicating bubble location relative to the system.

## 3 DISCUSSION

In explicit solvent MD simulations, proper solvation and system packing are essential to obtain realistic models and to reproduce accurate solute–solvent interactions. *Burbuja* provides an efficient solution for detecting and addressing low‐density voids across a wide range of system types and sizes, from non‐cubic water boxes to large viral assemblies.

We have demonstrated that *burbuja* can validate system preparation by detecting bubble formation early in the setup process and by tracking these voids during equilibration. This capability reduces the likelihood of artifacts during production simulations and helps prevent the unnecessary expenditure of computational resources on systems compromised by voids.

*Burbuja* also stands out from other tools such as VMD VolMap, which are not capable of tracking bubble formation or disappearance. While VMD VolMap facilitates the identification of voids through visualization of high‐density regions and can create data explorer file format (DX) density maps, *burbuja* can in addition, trigger an automatic output indicating whether bubbles are present. This feature makes *burbuja* suitable for use as an API to monitor the disappearance of bubbles during MD equilibration, as a validation of equilibration, and to detect the formation of bubbles during production runs, allowing simulations to be stopped at early stages to avoid unnecessary computational costs.

## 4 MATERIALS AND METHODS

### 4.1 Algorithm

**FIGURE 6:**
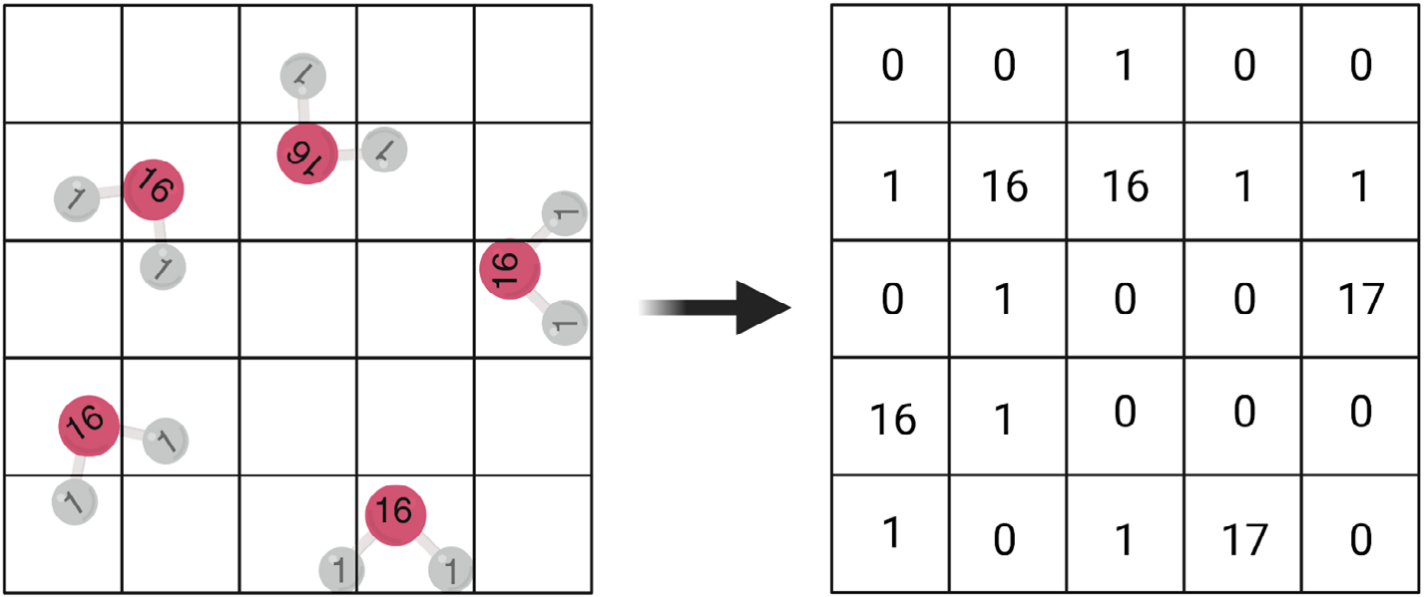
The mass of atoms falling within each grid cell (left) is used to calculate the corresponding voxel mass (right). This voxelization discretizes the simulation space, where each cell accumulates the total atomic mass it contains. The resulting coarse‐grained mass distribution provides the basis for computing local densities and identifying spatial density variations such as bubbles or other low‐density regions.

**FIGURE 7:**
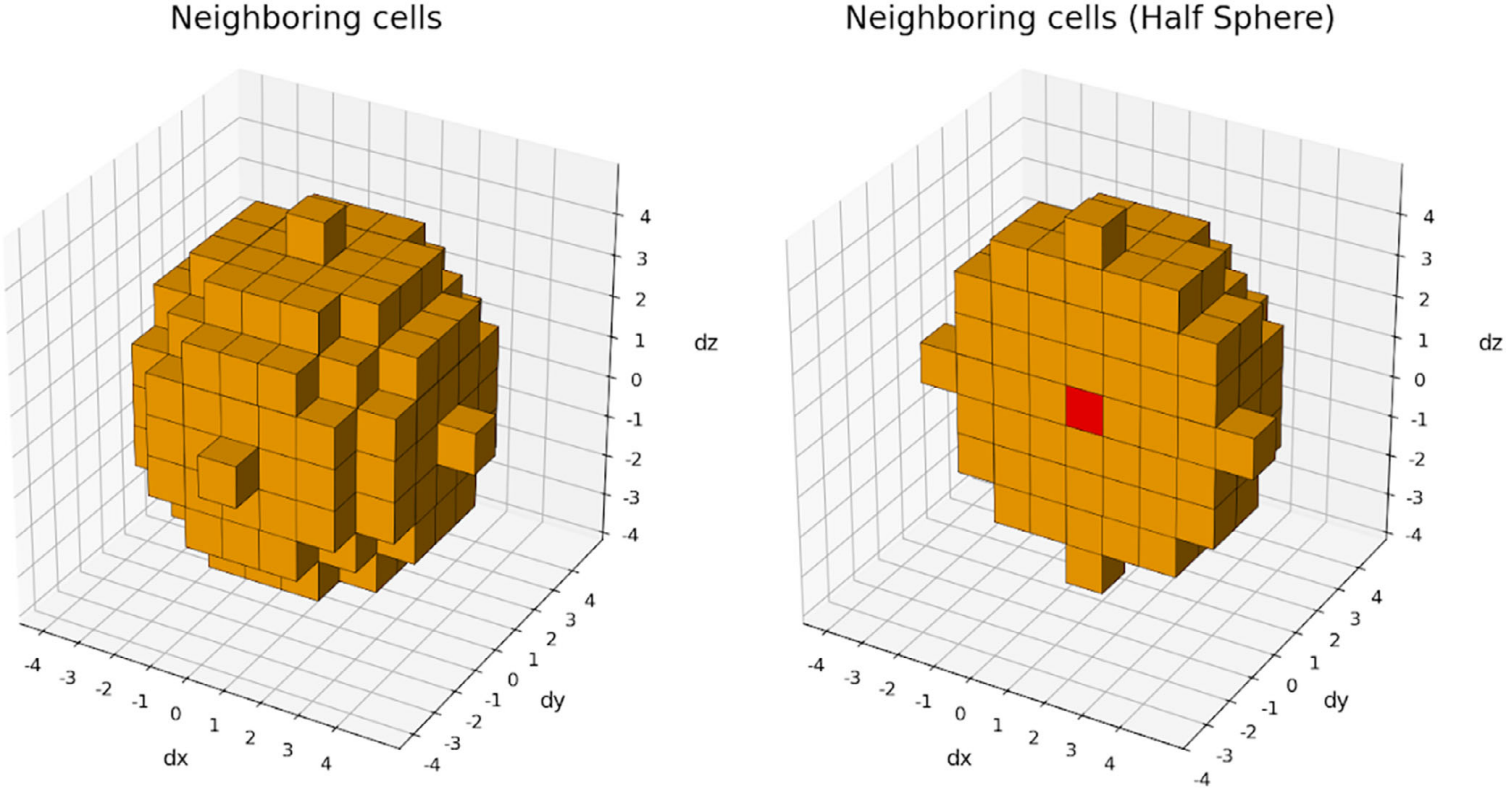
Orange cells (left) represent neighboring voxels within 4 Å that contribute to the density of the red central cell (right). This procedure enables a more meaningful estimate of local density, as density is a statistical property that cannot be accurately defined at the scale of individual atomic voxels.

*Burbuja* first wraps all atoms into a rectangular box—this procedure is applicable even for triclinic boundary systems—and then discretizes the space into a grid with a user‐defined resolution (1 Å by default). Each atom is assigned to its corresponding grid cell based on its coordinates, and the total mass within each cell is calculated (Figure 6). A neighbor‐averaged density for each cell is then computed by considering the mass and volume contributions of a spherical region encompassing neighboring cells. In its default configuration, *burbuja* includes neighbors up to 4 Å away in all directions (Figure 7).

Cells located near the grid edges include contributions from wrapped periodic images. Cells with density values below a defined cutoff (0.25 g/L by default) are classified as *voids*. If a single set of connected voids exceeds a defined volume (0.1 nm^3^ by default), *burbuja* designates the system as containing a bubble. The program can optionally characterize these bubbles by writing files for visualization and by reporting bubble shape and size statistics.

Default values such as neighbor distance, cutoff density values, and minimum bubble volumes were empirically chosen, as they seem to generate true positives and negatives while balancing performance for normal‐sized systems. However, in order to improve memory performance for the virion system, we had to increase the minimum bubble volume to 1 nm^3^. We anticipate that bubble detection and characterization should be relatively insensitive to the empirical choice of these parameters for the typical MD system.

### 4.2 Test systems

A diverse set of systems was used to test *burbuja*. These include systems containing intentional voids Figure 1, as well as systems without bubbles that nonetheless challenge the algorithm's ability to avoid false positives. Examples of the latter include simulations with non‐rectangular periodic boundary conditions, such as triclinic water boxes (Figure 3a), and systems in which proteins are positioned partially outside the solvent box (Figure 3b). The latter case produces artificial voids due to periodic boundary artifacts and therefore requires reshaping through the application of proper periodic wrapping (Figure 3b).

*Burbuja* was also tested on membrane‐containing systems (Figure 3c–e), which naturally feature low‐density regions within the lipid bilayer that can resemble bubbles. Moreover, tests on the severe acute respiratory syndrome corona virus 2 (SARS‐Cov‐2) M‐protein system (Figure 3d,e) demonstrate that *burbuja* works in CG models despite atoms that are grouped into single beads, resulting in less finely resolved densities. Additionally, the tool was evaluated using an MD equilibration trajectory of the trypsin–benzamidine system to assess its ability to track a transient void throughout minimization until its disappearance. Additionally, *burbuja* was applied to a large influenza virion system (~150 million atoms), where the assembly of protein and lipid substructures often results in poor packing and bubble formation. In order to avoid detecting very small bubbles, and to save memory and storage, we increased the minimum bubble volume to 10 nm^3^.

Finally, we also applied *burbuja* to a very large respiratory aerosol system equilibration trajectory (~1 billion atoms; five frames), which is prone to containing similar packing and bubble formation problems as the large virion system. Detecting and eliminating such artifacts during minimization and equilibration is essential to ensure accurate simulation of solvent–solute interactions. In order to detect only bubbles of a significant size, and in order to save memory, we increased the minimum bubble volume to 100 nm^3^, increased the grid resolution to 2 Å, and decreased the radius of the number of included neighboring voxel cells to 2.

## AUTHOR CONTRIBUTIONS

**Abraham Muñiz‐Chicharro:** Conceptualization; software; validation; visualization; writing – original draft; writing – review and editing. **Rommie E. Amaro:** Supervision; project administration; writing – review and editing. **Lane W. Votapka:** Conceptualization; writing – original draft; validation; visualization; writing – review and editing; software.

## CONFLICT OF INTEREST STATEMENT

The authors declare no conflicts of interest.

## Data Availability

*Burbuja* is open‐source and freely available at https://github.com/Abrahammc90/Burbuja.git. The repository also contains documentation, including online tutorials and Jupyter notebooks. The trypsin–benzamidine system and other unpublished test systems are also provided. Viral, aerosol, and CG structures were generously supplied by colleagues who performed related studies (Casalino et al., 2020, 2022).
